# Time-Limited Codewords over Band-Limited Channels: Data Rates and the Dimension of the W-T Space

**DOI:** 10.3390/e22090924

**Published:** 2020-08-23

**Authors:** Youssef Jaffal, Ibrahim Abou-Faycal

**Affiliations:** Department of Electrical and Computer Engineering, American University of Beirut, Beirut 1107 2020, Lebanon; Ibrahim.Abou-Faycal@aub.edu.lb

**Keywords:** information rates, degrees of freedom, band-limited, time-limited, prolate spheroidal wave functions

## Abstract

We consider a communication system whereby *T*-seconds time-limited codewords are transmitted over a *W*-Hz band-limited additive white Gaussian noise channel. In the asymptotic regime as WT→∞, it is known that the maximal achievable rates with such a scheme converge to Shannon’s capacity with the presence of 2WT degrees of freedom. In this work we study the degrees of freedom and the achievable information rates for finite values of WT. We use prolate spheroidal wave functions to obtain an information lossless equivalent discrete formulation and then we apply Polyanskiy’s results on coding in the finite block-length regime. We derive upper and lower bounds on the achievable rates and the corresponding degrees of freedom and we numerically evaluate them for sample values of 2WT. The bounds are asymptotically tight and numerical computations show the gap between them decreases as 2WT increases. Additionally, the possible decrease from 2WT in the available degrees of freedom is upper-bounded by a logarithmic function of 2WT.

## 1. Introduction

Wireless communication technologies that use radio waves impose tight requirements on the used spectrum as adjacent radio bands may be used by other users or technologies. A specific transmit spectral mask is usually required, a mask that allows the communication system to transmit data within a specific radio band while guaranteeing an acceptable level of interference on the other users or technologies. It is therefore reasonable to consider that in a wireless communication system the transmitter confines its transmitted data within its radio band, and the receiver looks for the transmitted data in this band. The allocated band, the corresponding channel statistics and the available transmit power determine the maximal reliable possible data rate. In his pioneering work, Shannon [1] derived the capacity of band-limited real additive white Gaussian noise (AWGN) channels, which for the complex channel is

(1) $$C_{Shannon}=2W\;\log_{2}\;\;\left[1+\frac{P}{2N_{0}W}\right]\;\;\mathrm{bits}\;\mathrm{per}\;\mathrm{seconds},$$

where *W* is the bandwidth of the baseband channel, *P* is the average transmit power and $N_{0}$ is the spectrum of the additive circularly symmetric complex white Gaussian noise. Shannon derived first the channel capacity for the discrete time channel as the block length of the codewords grows towards infinity. He then used the sampling theorem which provides a one-to-one relation between the continuous time and discrete time signals: For *W*-Hz band-limited signals when sampled at a rate of 2W samples per second, the operation is invertible and information lossless making use of the “sinc” function defined in this manuscript as

$$\mathrm{sinc}\left(2Wt\right)\;\overset{\mathrm{def}}{=}\;\frac{\sin\left(2\pi Wt\right)}{2\pi Wt}.$$

While the derived results are mathematically rigorous, some of the made assumptions do not hold in practical settings.

First: the conversion from discrete time to continuous time and vice versa is not practical since the “sinc” function needs an infinite time support. Moreover, the use of any band-limited function with non-zero finite energy is not possible in practice. In the literature, Wyner [2,3] and Gallager [4] tackled this issue and considered the use of *T*-seconds time-limited codewords. However, they derived asymptotic results as T→∞ and reached the same formula (1) derived by Shannon.

In [2], Wyner considered four different physical models and derived the asymptotic channel capacity for each model. Wyner stated that the first two models suffer from some physical difficulties; the assumed noise in the first model results in an infinite noise power at the receiver, and the use of strictly band-limited signals in the second model is not practical and may produce interference between consecutive codewords. On the other hand, he proved that using a noise model with finite power results in infinite capacity. Wyner made some assumptions to avoid these issues in [3] and in the third and fourth models in [2]. He derived the channel capacity of the different models by relating the continuous time to discrete time models as Shannon did in [1], but by using the prolate spheroidal wave functions (PSWFs) and their property that as 2WT→∞, the first 2WT PSWFs form asymptotically a complete orthonormal (CON) set for the time-limited and approximately band-limited signals.

Gallager [4] Section 8.5 considered transmitting time-limited signals over an additive real Gaussian noise channel with impulse response h(t). He used an arbitrary power spectral density (PSD) for the noise $S_{N}\left(f\right)$ and arbitrary filter h(t). As T→∞, he derived the channel capacity to be

$$C=\max_{p(f)}\int_{-\infty }^{\infty }\frac{1}{2}\;\log_{2}\;\;\left[1+\frac{p(f){\left|H(f)\right|}^{2}}{S_{N}\left(f\right)}\right]df,$$

where H(f) is the Fourier transform of the filter h(t) and p(f) is the transmit spectrum at frequency *f* subject to

$$\int_{-\infty }^{\infty }p\left(f\right)\;df\leq P.$$

In [4] Section 8.3 he considered the special case where H(f) is the ideal low pass filter and the noise is white and found the capacity to be the same as the one derived by Shannon, while avoiding the issues of infinite noise power and infinite capacity. In that special case (where H(f) is the ideal low pass filter) the transformation between continuous time and discrete time was also done through the use of the PSWFs [4] Sections 8.4 and 8.5. Finally, Gallager provided an intuitive argument regarding inter-codeword interference [4] Section 8.5; one can introduce a large guard time, say $T^{1-\epsilon }$ for some ϵ>0. Asymptotically, inter-codeword interference is avoided without affecting the data rates since $T^{1-\epsilon }/T\to 0$ as T→∞.

A related question is that of determining the degrees of freedom when using *T*-seconds time-limited codewords over a *W*-Hz band-limited channel. The space of finite-energy functions that are band-limited and time-limited contains only the zero function. It is nevertheless commonly accepted in the literature that the dimension of the W-T space is approximately 2WT. This argument is supported by results in [5,6,7,8,9] that were also derived in the asymptotic regime as WT→∞, something that we intend to relax hereafter.

Recently, in [10,11], we studied the use of *T*-seconds time-limited pulses over *W*-Hz real band-limited Gaussian channels, and derived the channel capacity by allowing the time duration of the codewords to grow towards infinity. In [10], we considered a pulse amplitude modulation (PAM) system and we studied optimal signaling; we showed that one can approach Shannon’s capacity by signaling at faster than the Nyquist rate. In [11], we considered a combined PAM-orthogonal multi-pulse modulation scheme (PAM-OMM) and derived the achievable rates and evaluated them numerically. We showed that these rates can be made arbitrarily close to the Shannon’s capacity by using a finite number of parallel filters. We also established that there are 2WT degrees of freedom when using such system.

Second: the second questionable assumption in practical settings is the use of infinite block-length codewords which is not feasible. In [12], Polyanskiy derived an approximation for the maximal data rates when given a target probability of error in the finite block-length regime, where only discrete time channels are considered.

In this work, we consider transmitting continuous time and finite duration codewords over a band-limited Gaussian channel. We use the ideal low pass filter as a model for the channel to force the transmitter to confine its transmitted information and energy in the allocated band and also model good receiver designs: Given any practical low pass filter, one can implement a sharper low pass filter that is closer to the ideal one. In such a model, there are no issues when it comes to infinite noise power and/or infinite capacity, however inter-codeword interference is inevitable.

Our main goal is to investigate the degrees of freedom and the achievable pairs of data rates and probability of error. We use a similar approach to [2,3,4] (using the PSWFs) to transform the problem from continuous time to discrete time and vice versa, and then we apply the adapted results by Polyanskiy for parallel discrete time AWGN channels.

Recently, in [13], we investigated the ‘dual’ problem where we derived an upper bound and a lower bound on the rates of source coding a *T*-seconds finite duration piece from a *W*-Hz band-limited real white Gaussian process.

The paper is organized as follows: in Section 2, we provide a brief overview of the PSWFs, some relevant properties in addition to some numerical computations. In Section 3, we present our system model and formulate the problem. We derive upper and lower bounds for the data rates and the corresponding degrees of freedom in Section 4. In Section 5, we present the results of our numerical computations. In Section 6, we present some possible enhancements on the bounds, and we summarize the results and conclude in Section 7.

## 2. Preliminaries: The Prolate Spheroidal Wave Functions

In [14], Slepian and Pollak showed that the PSWFs possess properties that make them useful in the Fourier analysis of band-limited functions and time-limited functions. For any c=πWT>0, they defined the PSWFs as an infinitely countable set of real functions ${\left\{\varphi_{c,l}\left(t\right)\right\}}_{l\in \mathbb{N}}$, normalized solutions of the integral equation where for every l∈ℕ,

$$\lambda_{c,l}\;\varphi_{c,l}\left(t\right)=\int_{\frac{-T}{2}}^{\frac{T}{2}}\frac{\sin2\pi W(t-s)}{\pi (t-s)}\varphi_{c,l}\left(s\right)\;ds,\;t\in \mathbb{R}.$$

The PSWFs form a CON set for band-limited functions [14] with

$$\int_{-\infty }^{\infty }\varphi_{c,l}\left(t\right)\varphi_{c,m}\left(t\right)\;dt=\delta_{lm},\;\forall \;l,m\in \mathbb{N}\times \mathbb{N},$$

where $\delta_{lm}$ is the Kronecker delta. Additionally, these functions are orthogonal over the time window *T*:

$$\int_{-\frac{T}{2}}^{\frac{T}{2}}\varphi_{c,l}\left(t\right)\varphi_{c,m}\left(t\right)\;dt=\lambda_{c,l}\;\delta_{lm},\;\forall \;l,m\in \mathbb{N}\times \mathbb{N},$$

where ${\left\{\lambda_{c,l}\right\}}_{l\in \mathbb{N}}$ are the eigenvalues that are all in the range $0<\lambda_{c,l}<1$ and decreasing in *l* [14]. The eigenvalue $\lambda_{c,l}$ may be hence viewed as the energy concentration of $\varphi_{c,l}\left(t\right)$ in the time interval $\left[-\frac{T}{2},\frac{T}{2}\right]$ and $\varphi_{c,0}\left(t\right)$ has the highest energy concentration. Additionally, the PSWFs are real continuous functions that are even when *l* is even and odd when *l* is odd.

In his book [4] Section 8.4, Gallager proved that the PSWFs are the desirable functions when sending time-limited signals over a band-limited channel. He made use of one important property of the PSWFs, namely the Fourier transform $\Phi_{c,l}\left(f\right)$ of the PSWF $\varphi_{c,l}\left(t\right)$ is a scaled version of a time-limited PSWF:

(2) $$\Phi_{c,l}\left(f\right)=\left\{\begin{array}{cc} j^{l}\sqrt{\frac{T}{2W\lambda_{c,l}}}\;\varphi_{c,l}\left(\frac{T}{2W}f\right) & \;\mathrm{for}\;f\in \left[-W,W\right]\\ 0 & \;\mathrm{otherwise} \end{array}\right.=j^{l}\sqrt{\frac{T}{2W\lambda_{c,l}}}\;\varphi_{c,l}\left(\frac{T}{2W}f\right)\mathrm{rect}\;\left(\;\frac{f}{2W}\;\right)$$

where $j\overset{\mathrm{def}}{=}\sqrt{-1}$.

In this paper we define $c\overset{\mathrm{def}}{=}2WT$ as index for the PSWFs, which is different from the one used by Slepian and Pollak. More specifically $\varphi_{1,l}\left(t\right)$ here is the same as $\varphi_{\frac{\pi }{2},l}\left(t\right)$ in [14]. We also denote here by “$D\varphi_{c,l}\left(t\right)$” the *T*-seconds time-limited version of a PSWF. More specifically,

(3) $$D\varphi_{c,l}\left(t\right)\overset{\mathrm{def}}{=}\varphi_{c,l}\left(t\right)\;\mathrm{rect}\;\left(\;\frac{t}{T}\;\right),$$

and since $\lambda_{c,l}$ is its energy, the normalized time-limited PSWF is $\frac{D\varphi_{c,l}\left(t\right)}{\sqrt{\lambda_{c,l}}}$. We denote by $\Phi_{c,l}^{D}\left(f\right)$ the Fourier transform of $\frac{D\varphi_{c,l}\left(t\right)}{\sqrt{\lambda_{c,l}}}$ which is equal to

(4) $$\Phi_{c,l}^{D}\left(f\right)=j^{l}\sqrt{\frac{T}{2W}}\;\varphi_{c,l}\left(\frac{T}{2W}f\right),\;f\in \mathbb{R}.$$

Based on (2)–(4), when $\frac{D\varphi_{c,l}\left(t\right)}{\sqrt{\lambda_{c,l}}}$ is passed through an ideal low pass filter with transfer function $\;\left(\;\frac{f}{2W}\;\right)$, the output is $\sqrt{\lambda_{c,l}}\;\varphi_{c,l}\left(t\right)$:

(5) $$\frac{D\varphi_{c,l}\left(t\right)}{\sqrt{\lambda_{c,l}}}\;*\;2W\mathrm{sinc}\left(2Wt\right)=\sqrt{\lambda_{c,l}}\;\varphi_{c,l}\left(t\right).$$

We have used a debugged version of the software package by Adelman et al. [15] to compute the eigenvalues of the PSWFs. While it is known [4] Section 8.4 that for any ϵ>0

$$\lim_{c\to \infty }\lambda_{c,(1+\epsilon )c}=0\;\&\;\lim_{c\to \infty }\lambda_{c,(1-\epsilon )c}=1,$$

**Figure 1:**
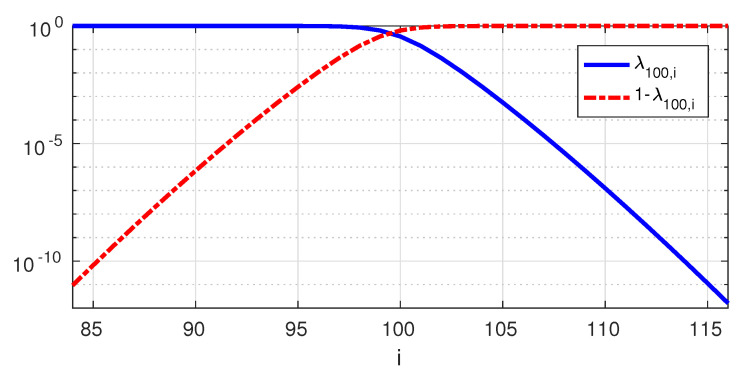
Eigenvalues of prolate spheroidal wave functions (PSWFs) for c=100 and 84≤l≤116.

Figure 1 shows the eigenvalues $\lambda_{c,l}$ of the PSWFs for c=100 and 84≤l≤116. Note that

$$\lambda_{100,l}<10^{-5}\;\mathrm{for}\;l>107,\;\&\;\lambda_{100,l}>1-10^{-5}\;\mathrm{for}\;l<92.$$

This transition region between the “extreme” eigenvalues (very close to 1 or 0) is known to have a length proportional to the logarithm of *c* [4] Section 8.4. For example, for c=2000, $\lambda_{2000,l}$ is between $10^{-5}$ and $1-10^{-5}$ only in the range 1988<l<2011.

In this manuscript, shifted (in time and frequency) PSWFs will come in handy. Denote by αc,l,mk,h the inner product between the $l^{th}$ normalized time-limited PSWF already shifted in time and frequency by kT-seconds and h2W-Hz, respectively, and the band-limited version of the $m^{th}$ normalized time-limited PSWF. By Equation (5) and using Parseval,

(6) αc,l,mk,h=def〈Dφc,l(t−kT)λc,lej2πh2W(t−kT),λc,mφc,m(t)〉=〈Φc,lD(f−h2W)e−j2πkTf,Φc,mD(f)rectf2W〉=〈Φc,lD(f−h2W)e−j2πkTfrectf2WΦc,mD(f)〉,

which can be interpreted as the inner product between the bandlimited shifted normalized $l^{th}$ PSWF and the $m^{th}$ normalized time-limited PSWF. In Appendix A, we study the magnitude of αc,l,mk,h and derive various bounds that are used throughout this document.

## 3. System Model and Problem Formulation

**Figure 2:**
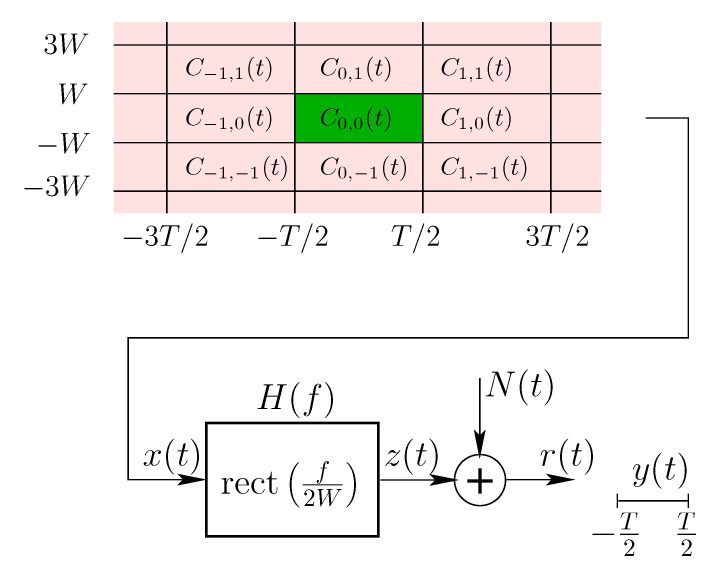
Continuous time system model.

We consider a system model where a *T*-seconds time-limited codeword is transmitted over a linear Gaussian channel with transfer function H(f)—assumed to be an ideal *W*-Hz low-pass filter, and an additive complex Gaussian noise N(t), assumed to be a stationary *W*-Hz band-limited “white” process with mean zero and PSD $S_{N}\left(f\right)=N_{o}$ for f∈[−W,W]. With a system in mind whereby other codewords may be transmitted—possibly by other users—consecutively and/or in neighboring bands, we denote by $C_{0,0}\left(t\right)$ the codeword carrying the data packet of interest, and by ${\left\{C_{k,h}\left(t\right)\right\}}_{\left(k,h\right)\in {\mathbb{Z}}^{2}\backslash \left\{\left(0,0\right)\right\}}$ those carrying other data packets, possibly transmitted by other devices and interfering with the message of interest as illustrated in Figure 2. Our model is based on the reasonable assumption that all codewords follow the same modulation techniques, since every frequency band is usually allocated to a unique technology which abides by specific standards, and the neighboring bands are more likely to be used by the same technology.

In what follows, we consider various scenarios where some or all of those interfering codewords are present and we denote by $I\subset {\mathbb{Z}}^{2}\backslash \left\{\left(0,0\right)\right\}$ the set of other present codewords. The overall signal going through the channel can hence be written as the sum of the codeword of interest and the other interfering codewords:

$$x\left(t\right)=C_{0,0}\left(t\right)+\sum_{(k,h)\in I}C_{k,h}\left(t\right),$$

where $C_{k,h}\left(t\right)$ is non-zero only over t∈[−T/2+kT,T/2+kT]. On the receiver side, the data packet of interest is to be recovered from y(t),t∈[−T/2,T/2], a *T*-seconds time-limited version of the output of the channel r(t).

We assume that, whenever present, a transmitted codeword satisfies the power constraint,

(7) $$\frac{1}{T}\int_{\frac{-T}{2}}^{\frac{T}{2}}|C_{k,h}\left(t+kT\right){|}^{2}dt\leq P.$$

As Gallager proved that the PSWFs are the desirable CON set when sending a time-limited codeword over a band-limited channel [4] Section 8.4, we use the normalized time-limited PSWF as orthogonal pulses to send the data symbols. Hence the codewords can be written as

$$C_{k,h}\left(t\right)=\sum_{l=0}^{\infty }a_{k,h,l}\frac{D\varphi_{c,l}\left(t-kT\right)}{\sqrt{\lambda_{c,l}}}\;e^{j2\pi h\;2W(t-k\;T)}\;-\mathrm{non-zero}\;\mathrm{only}\;\mathrm{on}\;t\in \left[-\frac{T}{2}+kT,\frac{T}{2}+kT\right],$$

where c=2WT. Representing the continuous time signal $C_{k,h}\left(t\right)$ by the symbols $\left\{a_{k,h,l}\right\}$ is known as “signal space representation” in the context of digital communications. The $\left\{a_{k,h,l}\right\}$’s are chosen from a given complex signal constellation and by Plancherel and (7) they satisfy

(8) $$\frac{1}{T}\;\sum_{l=0}^{\infty }{\left|a_{k,h,l}\right|}^{2}=\frac{1}{T}\int_{\frac{-T}{2}}^{\frac{T}{2}}|{C_{k,h}\left(t+kT\right)|}^{2}dt\leq P.$$

When it comes to the noise, N(t) is band-limited and can be hence decomposed as

$$N\left(t\right)=\sum_{l=0}^{\infty }n_{l}\;\varphi_{c,l}\left(t\right),$$

where ${\left\{n_{l}\right\}}_{l\in \mathbb{Z}}$ are independent zero-mean complex circular Gaussian random variables with variance $N_{0}$.

At the receiver, sufficient statistics are clearly obtained by projecting y(t) on the set of normalized time-limited PSWF to extract the data symbols. It is worth noting that since r(t) is band-limited and has finite energy, it is necessarily analytic and it is therefore sufficient to know r(t) over any open interval to determine it fully. As a consequence, from an information-theoretic perspective, whether r(t) as whole is available or only y(t), the information rates are identical.

The problem at hand is to maximize the information rates given a maximal probability of error. This is naturally related to the available degrees of freedom when sending time-limited codewords over a band-limited channel, which is the maximal number of independent data symbols that can be transmitted to the receiver.

In the following section, we consider various scenarios and derive upper and lower bounds for the data rates and the degrees of freedom.

## 4. Bounds on the Data Rates

### 4.1. An Upper Bound

**Figure 3:**
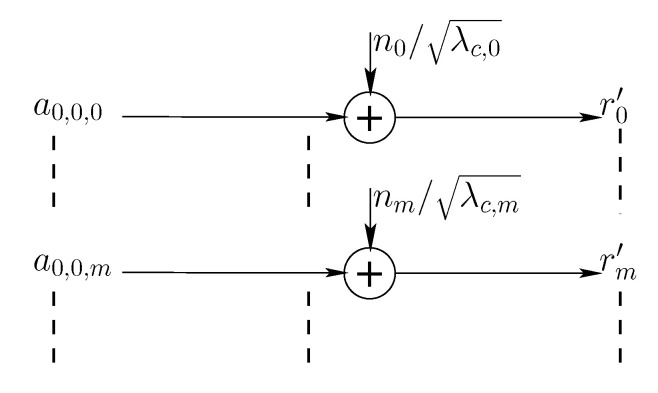
Equivalent discrete time system model.

To derive an upper bound, we consider the case where only $C_{0,0}\left(t\right)$ is transmitted over the channel. By ignoring the other transmitted codewords we ignore the effect of inter-codeword interference, and we obtain upper bounds on the rates and the degrees of freedom since interference can only be harmful. In this scenario, the input to the channel can be written as

$$x\left(t\right)=\sum_{m=0}^{\infty }a_{0,0,m}\frac{D\varphi_{c,m}\left(t\right)}{\sqrt{\lambda_{c,m}}},$$

and the received signal r(t) is band-limited and can be written as

$$\begin{array}{cc} \; & r\left(t\right)=\sum_{m=0}^{\infty }a_{0,0,m}\sqrt{\lambda_{c,m}}\varphi_{c,m}\left(t\right)+N\left(t\right)=\sum_{m=0}^{\infty }\left[a_{0,0,m}\sqrt{\lambda_{c,m}}+n_{m}\right]\varphi_{c,m}\left(t\right)\\ \Rightarrow \; & y\left(t\right)=r\left(t\right)\;\mathrm{rect}\;\left(\;\frac{t}{T}\;\right)=\sum_{m=0}^{\infty }\left[a_{0,0,m}\sqrt{\lambda_{c,m}}+n_{m}\right]D\varphi_{c,m}\left(t\right)=\sum_{m=0}^{\infty }y_{m}\;D\varphi_{c,m}\left(t\right), \end{array}$$

where $y_{m}=\sqrt{\lambda_{c,m}}\;a_{0,0,m}+n_{m}$. From an information theoretic perspective, the considered system model is equivalent to the discrete time system model in Figure 3 where $r_{m}^{\prime }=y_{m}/\sqrt{\lambda_{c,m}}=a_{0,0,m}+n_{m}/\sqrt{\lambda_{c,m}}$.

The noise components $\left\{n_{m}/\sqrt{\lambda_{c,m}}\right\}$ are independent, zero-mean complex circular Gaussian random variables and each complex channel is equivalent to two usages of independent real (real and imaginary) channels with additive Gaussian noise with variance $N_{0}/2\lambda_{c,m}$ per dimension. If $𝖤\left[|a_{0,0,m}{|}^{2}\right]=2P_{m}T$ (where $P_{m}T$ is the second moment per dimension), the power constraint in (8) can be written as $2\sum_{m=0}^{\infty }P_{m}\leq P$ and the signal to noise ratio per dimension for $r_{m}^{\prime }$ is $\frac{2\lambda_{c,m}P_{m}T}{N_{0}}$.

One can notice that it is possible to send infinitely many independent symbols in such a system. However, only a finite number of them, say *L*, is useful because the energy per symbol is finite and the noise energy of the $m^{\mathrm{th}}$ channel is increasing towards infinity as *m* increases to infinity ($\lambda_{c,m}$ tends to 0 as *m* tends to *∞* as shown in Section 2). Note that *L* depends on c=2WT since there are approximately *c* eigenvalues $\lambda_{c,m}$ that are close to 1, and naturally *L* grows to infinity with *c*.

Polyanskiy [12] derived upper and lower bounds and an approximation for the achievable rates at a given probability of error in the finite block-length regime. In [16], he studied the parallel Gaussian channel set-up where *N* memoryless parallel channels of different noise power are used each *n* times. Following the methodology in [12] and applying the Berry–Esseen inequality [12] Lemma 14 over n×N real independent variables shows that the maximal number of bits that can be transmitted is

(9) $$\begin{array}{c} \frac{n}{2}\sum_{m=0}^{N-1}\log_{2}\;\;\left[1+\frac{2\lambda_{c,m}P_{m}T}{N_{0}}\right]-\sqrt{n\sum_{m=0}^{N-1}V_{1}\;\;\left[\frac{2\lambda_{c,m}P_{m}T}{N_{0}}\right]}Q^{-1}\left(\epsilon \right)+O\left(\log_{2}\left(nL\right)\right)\;\mathrm{bits}, \end{array}$$

where

- ϵ∈(0,1) is the probability of error,
- $V_{1}\left[\theta \right]=\frac{\theta }{2}\frac{\theta +2}{{\left(\theta +1\right)}^{2}}\log_{2}^{2}e$,
- $\left\{P_{m}\right\}$ is the water-filling solution such that $P_{m}={\left[\mu -\frac{N_{0}}{2\lambda_{c,m}T}\right]}^{+}$ and $n\sum_{m=0}^{N-1}P_{m}=P$,
- and *L* is the number of non-zero $\left\{P_{m}\right\}$’s, which is less or equal *N*.

In Polyanskiy’s work [16] *N* is constant and *n* grows towards infinity and hence [16] Theorem 4 shows an $O(\log_{2}n)$ term instead of the $O(\log_{2}nL)$ term here in (9). In our setup, each channel *m* in Figure 3 has a different noise power, but each has two dimensions with the same noise power. In our scenario therefore n=2, *L* is of the order of (and grows with) *c* and the term $O(\log_{2}nL)$ becomes $O(\log_{2}L)$ as *n* is constant and equal to 2. These derivations yield the following lemma:

**Lemma** **1.** *An upper bound on the data rates is given by,*

(10) $$\begin{array}{cc} R_{UB}\left(\epsilon ,P,c\right) & =\frac{1}{T}\sum_{m=0}^{\infty }\log_{2}\;\;\left[1+\frac{2\lambda_{c,m}P_{m}T}{N_{0}}\right]-\frac{1}{T}\sqrt{2\sum_{m=0}^{\infty }V_{1}\;\;\left[\frac{2\lambda_{c,m}P_{m}T}{N_{0}}\right]}Q^{-1}\left(\epsilon \right)+\frac{1}{T}O\left(\log_{2}\left(L\right)\right)\;b/s.\\ \; & =\frac{1}{T}\sum_{m=0}^{L-1}\log_{2}\;\;\left[1+\frac{2\lambda_{c,m}P_{m}T}{N_{0}}\right]-\frac{1}{T}\sqrt{2\sum_{m=0}^{L-1}V_{1}\;\;\left[\frac{2\lambda_{c,m}P_{m}T}{N_{0}}\right]}Q^{-1}\left(\epsilon \right)+\frac{1}{T}O\left(\log_{2}\left(L\right)\right)\;b/s. \end{array}$$

*where*

- *ϵ∈(0,1) is the probability of error,*
- *$V_{1}\left[\theta \right]=\frac{\theta }{2}\frac{\theta +2}{{\left(\theta +1\right)}^{2}}\log_{2}^{2}e$,*
- *$\left\{P_{m}\right\}$ is the water-filling solution such that $P_{m}={\left[\mu -\frac{N_{0}}{2\lambda_{c,m}T}\right]}^{+}$ and $2\sum_{m=0}^{\infty }P_{m}=2\sum_{m=0}^{L-1}P_{m}=P$,*
- *and L is the number of non-zero $\left\{P_{m}\right\}$s.*

### 4.2. Lower Bounds

In what follows, we derive a lower bound on the rates by jointly:

• Finding an upper bound on the interference.
• Using only the first *N* PSWFs to transmit data.

Subsequently, we optimize over the value of *N* to obtain tighter bounds as well as lower bounds on the degrees of freedom.

We study below three scenarios for the interference and derive a lower bound for these scenarios.

#### 4.2.1. Consecutive Single Band Codewords (CSB)

**Figure 4:**
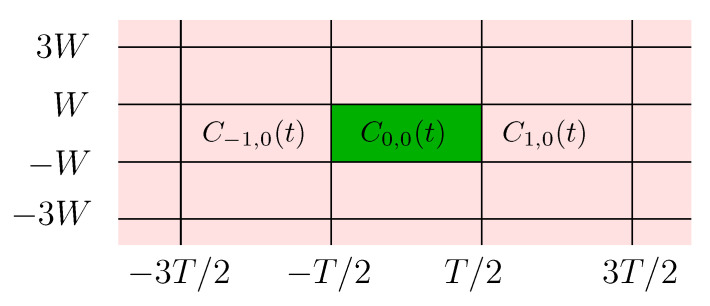
Single band interference.

We consider first the case where a single band is used and only the codewords ${\left\{C_{k,0}\left(t\right)\right\}}_{k\in \mathbb{Z}}$ are transmitted over the channel, i.e., $I=\{\left(k,0\right),k\in {\mathbb{Z}}^{*}\overset{\mathrm{def}}{=}\mathbb{Z}\backslash \left\{0\right\}\}$ as shown in Figure 4.

In the case where only the first *N* PSWFs are used,

$$x\left(t\right)=C_{\left(0,0\right)}\left(t\right)+\sum_{k\in {\mathbb{Z}}^{*}}C_{k,0}\left(t\right)=\sum_{l=0}^{N-1}a_{0,0,l}\frac{D\varphi_{c,l}\left(t\right)}{\sqrt{\lambda_{c,l}}}+\sum_{k\in {\mathbb{Z}}^{*}}\sum_{l=0}^{N-1}a_{k,0,l}\frac{D\varphi_{c,l}\left(t-kT\right)}{\sqrt{\lambda_{c,l}}},$$

and the received signal is the sum of an information bearing signal, an interfering signal and additive channel noise,

$$r\left(t\right)=\left[\sum_{l=0}^{N-1}a_{0,0,l}\sqrt{\lambda_{c,l}}\;\varphi_{c,l}\left(t\right)\right]+\left[\sum_{k\in {\mathbb{Z}}^{*}}\sum_{l=0}^{N-1}a_{k,0,l}\sqrt{\lambda_{c,l}}\;\varphi_{c,l}\left(t-kT\right)\right]+N\left(t\right).$$

Projecting r(t) on the $m^{th}$ normalized time-limited PSWF—which can be done using an appropriate matched filter—results in

$$\begin{array}{c} y_{m}=\lambda_{c,m}\;a_{0,0,m}+w_{m}, \end{array}$$

where the interference plus noise term $w_{m}$ is

wm=∑k∈ℤ*∑l=0N−1αc,l,mk,0ak,0,l+λc,mnm,

where αc,l,mk,h is defined in Equation (6). Next, we upper bound its second moment,

𝖤|wm|2=𝖤|∑k∈ℤ*∑l=0N−1αc,l,mk,0ak,0,l|2+λc,mN0=∑k∈ℤ*𝖤|∑l=0N−1αc,l,mk,0ak,0,l|2+λc,mN0.

Since the ${\left\{a_{k,0,l}\right\}}_{l=0}^{N-1}$ are not necessarily uncorrelated for a fixed *k*, we use the upper bound

(11) $$|\sum_{l=1}^{N}c_{l}{|}^{2}\leq N\sum_{l=1}^{N}{|c_{l}|,}^{2}$$

and the fact that $𝖤\left[|a_{k,0,l}{|}^{2}\right]=2P_{l}T$ to upper bound the second moment

𝖤|wm|2≤∑k∈ℤ*N∑l=0N−1|αc,l,mk,0|2𝖤|ak,0,l|2+λc,mN0=N∑l=0N−12PlT∑k∈ℤ*|αc,l,mk,0|2+λc,mN0.

Using the bound (A11) derived in Appendix A

∑k∈ℤ*|αc,l,mk,0|2≤λc,l(1−λc,l)⟹𝖤|wm|2≤N∑l=0N−12PlTλc,l(1−λc,l)+λc,mN0.

Using the alternative bound (A7) and the fact that $\sum_{l=0}^{N-1}2P_{l}T\leq PT$,

∑k∈ℤ*|αc,l,mk,0|2≤λc,m(1−λc,m)⟹𝖤|wm|2≤λc,mN(1−λc,m)PT+N0.

Therefore the second moment of the interference term is upper-bounded by

(12) $$I_{CSB}\left[m\right]\overset{\mathrm{def}}{=}\min\left(N\sum_{l=0}^{N-1}2P_{l}T\;\lambda_{c,l}\left(1-\lambda_{c,l}\right)\;,\;\lambda_{c,m}N\left(1-\lambda_{c,m}\right)PT\right),$$

and the signal to noise and interference ratio per dimension in $y_{m}$ is lower-bounded by

$$S_{CSB}\left[m\right]\overset{\mathrm{def}}{=}\frac{\lambda_{c,m}^{2}2P_{m}T}{I_{CSB}\left[m\right]+\lambda_{c,m}N_{0}}.$$

These derivations yield the following lemma:

**Lemma** **2.** *A lower bound on the data rates is given by*

(13) $$R_{CSB}\left(\epsilon ,P,c\right)=\max_{N,{\left\{P_{m}\right\}}_{m}}\frac{1}{T}\left[\sum_{m=0}^{N-1}\log_{2}\;\;\left[1+S_{CSB}\left[m\right]\right]-\sqrt{2\sum_{m=0}^{N-1}V_{1}\;\;\left[S_{CSB}\left[m\right]\right]}Q^{-1}\left(\epsilon \right)+O\left(\log_{2}\left(L\right)\right)\right],$$

*where L is the number of non-zero $\left\{P_{m}\right\}$s.*

#### 4.2.2. Single Time-Slot Multi-Band Codewords (STMB)

**Figure 5:**
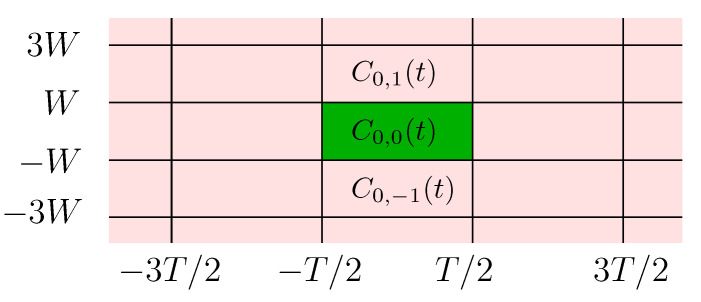
Single time-slot interference.

In this scenario multiple bands in a single time-slot are used and only the codewords ${\left\{C_{0,h}\left(t\right)\right\}}_{h\in \mathbb{Z}}$ are transmitted over the channel, i.e., $I=\{\left(0,h\right),h\in {\mathbb{Z}}^{*}\}$ as shown in Figure 5 below.

As above, the output of the $m^{th}$ matched filter is

$$y_{m}=\lambda_{c,m}\;a_{0,0,m}+w_{m},$$

where the interference plus noise term is now

wm=∑h∈ℤ*∑l=0N−1αc,l,m0,ha0,h,l+λc,mnm.

Using the bound (A12), one can upper bound the second moment of $w_{m}$,

∑h∈ℤ*|αc,l,m0,h|2≤λc,m2(1−λc,m)λc,l⟹𝖤|wm|2=𝖤|∑h∈ℤ*∑l=0N−1αc,l,m0,ha0,h,l|2+λc,mN0≤λc,m2(1−λc,m)N∑l=0N−12PlTλc,l+λc,mN0.

Alternatively, using the bound (A8)

∑h∈ℤ*|αc,l,m0,h|2≤λc,m(1−λc,l)⟹𝖤|wm|2=𝖤|∑h∈ℤ*∑l=0N−1αc,l,m0,ha0,h,l|2+λc,mN0≤λc,mN∑l=0N−1(1−λc,l)2PlT+λc,mN0.

Therefore the second moment of the interference term is upper-bounded by

(14) $$I_{STMB}\left[m\right]\overset{\mathrm{def}}{=}\min\left(\lambda_{c,m}^{2}\left(1-\lambda_{c,m}\right)N\sum_{l=0}^{N-1}\frac{2P_{l}T}{\lambda_{c,l}}\;,\;\lambda_{c,m}N\sum_{l=0}^{N-1}\left(1-\lambda_{c,l}\right)2P_{l}T\right).$$

These derivations yield the following lemma:

**Lemma** **3.** *The corresponding lower bound on the data rates is given by*

(15) $$R_{STMB}\left(\epsilon ,P,c\right)=\max_{N,{\left\{P_{m}\right\}}_{m}}\frac{1}{T}\left[\sum_{m=0}^{N-1}\log_{2}\;\;\left[1+S_{STMB}\left[m\right]\right]-\sqrt{2\sum_{m=0}^{N-1}V_{1}\;\;\left[S_{STMB}\left[m\right]\right]}Q^{-1}\left(\epsilon \right)+O\left(\log_{2}\left(L\right)\right)\right],$$

*where L is the number of non-zero $\left\{P_{m}\right\}$’s and*

$$S_{STMB}\left[m\right]=\frac{\lambda_{c,m}^{2}2P_{m}T}{I_{STMB}\left[m\right]+\lambda_{c,m}N_{0}}.$$

#### 4.2.3. Consecutive Multi-Band Codewords (CMB)

We consider now the case where all the codewords ${\left\{C_{k,h}\right\}}_{\left(k,h\right)\in {\mathbb{Z}}^{2}}$ are transmitted over the channel. The analysis follows as above and the interference plus noise term $w_{m}$ is

wm=∑(k,h)∈ℤ2\(0,0)∑l=0N−1αc,l,mk,hak,h,l+λc,mnm=∑k∈ℤ*∑l=0N−1αc,l,mk,0ak,0,l+∑h∈ℤ*∑l=0N−1αc,l,m0,ha0,h,l+∑(k,h)∈ℤ*×ℤ*∑l=0N−1αc,l,mk,hak,h,l+λc,mnm.

Upper bounds on the second moments of the first two interference terms have been derived in Section 4.2.1 and Section 4.2.2, respectively, and it remains to derive one for the third term. By Equation (11),

𝖤|∑k∈ℤ*∑h∈ℤ*∑l=0N−1αc,l,mk,hak,h,l|2≤N∑l=0N−1𝖤|∑h∈ℤ*∑k∈ℤ*ak,h,lαc,l,mk,h|2=N∑l=0N−1∑h∈ℤ*∑k∈ℤ*𝖤|ak,h,l|2|αc,l,mk,h|2≤N∑l=0N−12PlT∑k∈ℤ*∑h∈ℤ*|αc,l,mk,h|2.

Using bound (A13) and Equations (12) and (14),

$$\begin{array}{c} 𝖤\left[|w_{m}{|}^{2}\right]\leq I_{CSB}\left[m\right]+I_{STMB}\left[m\right]+N\sum_{l=0}^{N-1}\left(1-\lambda_{c,l}\right)2P_{l}T+\lambda_{c,m}N_{0} \end{array}$$

Alternatively, using bound (A14),

$$\begin{array}{c} 𝖤\left[|w_{m}{|}^{2}\right]\leq I_{CSB}\left[m\right]+I_{STMB}\left[m\right]+N\sum_{l=0}^{N-1}\frac{\lambda_{c,m}}{\lambda_{c,l}}\left(1-\lambda_{c,m}\right)2P_{l}T+\lambda_{c,m}N_{0}, \end{array}$$

Therefore, the second moment on the interference terms is upper-bounded by

$$I_{CMB}\left[m\right]\overset{\mathrm{def}}{=}I_{CSB}\left[m\right]+I_{STMB}\left[m\right]+\min\left(N\sum_{l=0}^{N-1}\left(1-\lambda_{c,l}\right)2P_{l}T\;,\;N\sum_{l=0}^{N-1}\frac{\lambda_{c,m}}{\lambda_{c,l}}\left(1-\lambda_{c,m}\right)2P_{l}T\right).$$

These derivations yield the following lemma:

**Lemma** **4.** *The corresponding lower bound on the data rates is given by*

(16) $$R_{LB}\left(\epsilon ,P,c\right)=\max_{N,{\left\{P_{m}\right\}}_{m}}\frac{1}{T}\left[\sum_{m=0}^{N-1}\log_{2}\;\;\left[1+S_{CMB}\left[m\right]\right]-\sqrt{2\sum_{m=0}^{N-1}V_{1}\;\;\left[S_{CMB}\left[m\right]\right]}Q^{-1}\left(\epsilon \right)+O\left(\log_{2}\left(L\right)\right)\right],$$

*where L is the number of non-zero $\left\{P_{m}\right\}$s and*

$$S_{CMB}\left[m\right]\overset{\mathrm{def}}{=}\frac{\lambda_{c,m}^{2}2P_{m}T}{I_{CMB}\left[m\right]+\lambda_{c,m}N_{0}}.$$

## 5. Numerical Results

In what follows, we use W=1 KHz and $\epsilon =10^{-3}$ in our computations. Since we consider the complex base-band channel, an equivalent real pass-band channel will have 2W-Hz bandwidth (i.e., 2 KHz in our case), and *L* complex degrees of freedom in base-band is equivalent to 2L real degrees of freedom in passband.

In the following we evaluate the degrees of freedom and the data rates for different 𝖲𝖭𝖱 levels (in dB) as *L* depends on the 𝖲𝖭𝖱; for example, since the water-filling algorithm is used for Equation (10), *L* can be increased by increasing the “water level” in the water-filling algorithm, which means that the 𝖲𝖭𝖱 level must be increased. Note that for a fixed *c*, the ratio of the derived bounds to Shannon’s capacity depends only on $P,N_{o}$ and *W* through the ratio $\frac{P}{N_{o}W}$, and the results for W≠1 KHz can be extracted from our presented results for an appropriate range of 𝖲𝖭𝖱 $=2P/N_{o}$.

To evaluate the bounds in Equations (10), (13), (15) and (16), we used the optimization toolbox in MATLAB to search for the optimal solution in ${\left\{P_{m}\right\}}_{m}$. When it comes to the upper bound (10), the water-filling choice used by Polyanskiy only maximizes the term $\sum_{m=0}^{\infty }\log_{2}\;\left[1+\frac{2\lambda_{c,m}P_{m}T}{N_{0}}\right]$ and not the whole expression. However, the water-filling choice almost achieves the same performance as the optimization routine with negligible differences. When it comes to the term $O(\log_{2}\left(L\right))$, we specialize it to $\frac{1}{2}\log_{2}\left(2L\right)$ for the sake of numerical computations; we used the constant $\frac{1}{2}$ as Polyanskiy conjectured in Equation (4.218) in his thesis [12], and we used the term 2L inside $\log_{2}\left(.\right)$ since the number of real independent variables is 2L (as explained in Section 4.1).

### 5.1. Upper Bound

To solve Equation (10), we maximize the following quantity using the optimization tool in MATLAB

$$\begin{array}{cc} R_{UB}^{N}= & \max_{P_{m}}\frac{1}{T}\sum_{m=0}^{N-1}\log_{2}\;\;\left[1+\frac{2\lambda_{c,m}P_{m}T}{N_{0}}\right]-\frac{1}{T}\sqrt{2\sum_{m=0}^{N-1}V_{1}\;\;\left[\frac{2\lambda_{c,m}P_{m}T}{N_{0}}\right]}Q^{-1}\left(\epsilon \right), \end{array}$$

over ${\left\{P_{m}\right\}}_{m}^{N-1}$ for different values of *N*. The solution being decreasing with *m*, *L* is the value of *N* where $R_{UB}^{N}$ saturates (which is the same as the number of non-zero $P_{m}$’s after saturation). We adopt similar method and notations for all the bounds presented hereafter.

**Figure 6:**
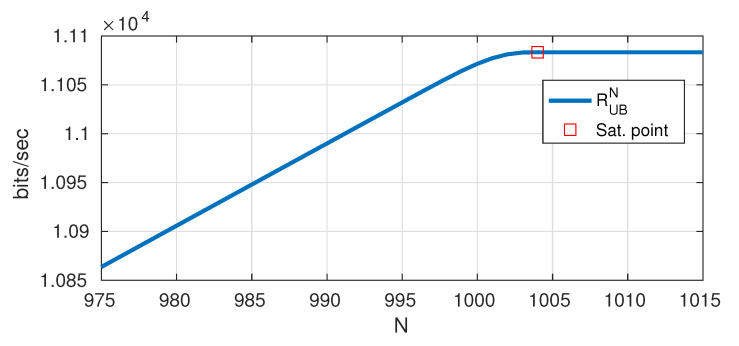
Saturation of $R_{UB}^{N}$ for c=1000 and 𝖲𝖭𝖱 =50 dB.

Figure 6 shows the obtained $R_{UB}^{N}$ for c=1000 and at 𝖲𝖭𝖱 =50 dB, and in this example the obtained degrees of freedom are L=1004. The upper bound on the rates is

$$R_{UB}\left(\epsilon ,P,c\right)=R_{UB}^{L}+\frac{1}{2T}\log_{2}\left(2L\right)\;\mathrm{bits}/\sec.$$

**Figure 7:**
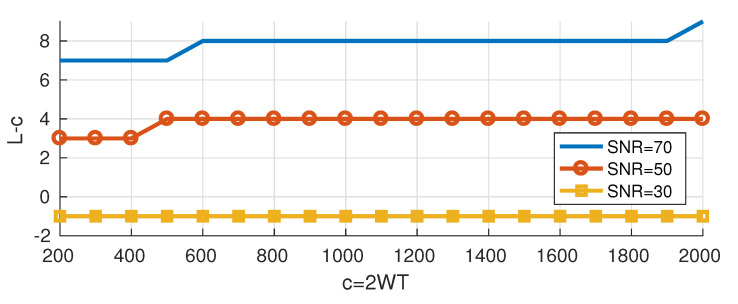
Upper bounds on the degrees of freedom.

We compute *L* and the corresponding upper bound $R_{UB}$ for different values of *c* and for different levels of 𝖲𝖭𝖱. Figure 7 shows the difference (L−c) between the obtained degrees of freedom and c=2WT, and as expected, *L* increases as the 𝖲𝖭𝖱 increases (in a manner akin to the water-filling solution: as the water level increases, it is possible to use additional PSWFs). However, the additional degrees of freedom (beyond c) increase slowly with *c* and L/c→1 as *c* increases towards infinity.

**Figure 8:**
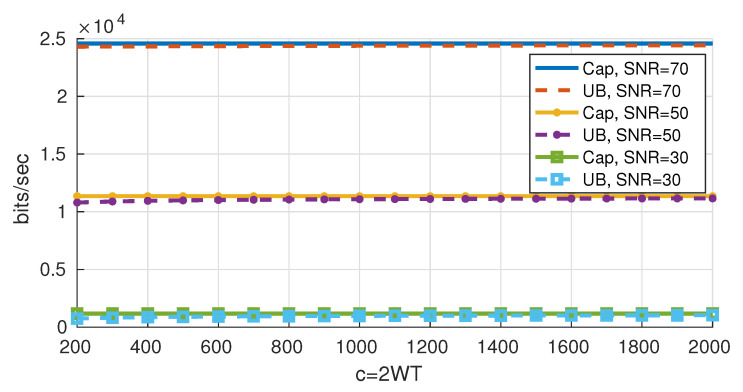
Upper bounds on the data rates.

In Figure 8 we plot the obtained upper bound on the rates together with the Shannon capacity. We notice that the gap between the upper bounds and the Shannon capacity decreases as *c* increases.

The ratio of the bounds to the Shannon capacity can be seen in Figure 10 below.

### 5.2. Lower Bounds

In this section, we present the numerical results for Section 4.2.1, Section 4.2.2 and Section 4.2.3 (Equations (13), (15) and (16)), and we apply the same numerical method we used in the previous section. We notice that the lower bounds when using either CSB or STMB are almost the same with no significant differences and we omit the results for CSB.

**Figure 9:**
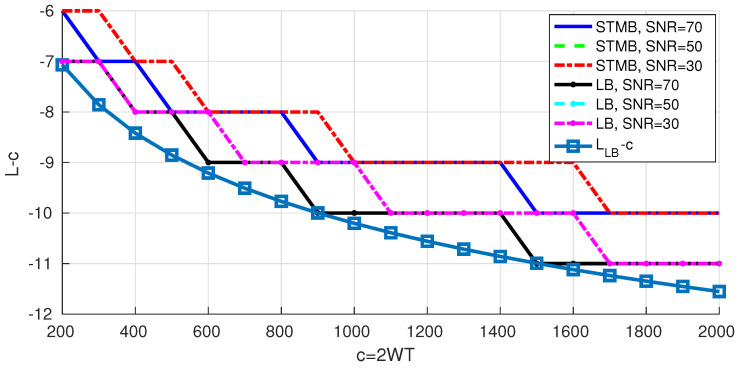
Lower bounds on the degrees of freedom.

Figure 9 shows the obtained lower bounds on the degrees of freedom. We note that for given scenario (CSB, STMB or the general lower bound), the results are almost the same for different 𝖲𝖭𝖱 levels; increasing the signal power will only lead to increasing the power of the interference (see Equations (13), (15) and (16)), and the effect on the signal to interference ratio remains negligible. Moreover, the results for the different scenarios are very close (±1 on average). Although L−c decreases as *c* increases, it decreases slowly and L/c→1 as *c* increases towards infinity.

We propose approximating the degrees of freedom for the general lower bound by the following equation

(17) $$L_{LB}\;\hat{=}\;c-1.35\log_{2}\left(c\right)+3.25,$$

and we draw “$L_{LB}-c$” in Figure 9. It is expected that “$L_{LB}-c$” is a logarithmic function of *c* since the transition region of the eigenvalues of PSWFs is a logarithmic function of *c* (as shown in Section 2).

**Figure 10:**
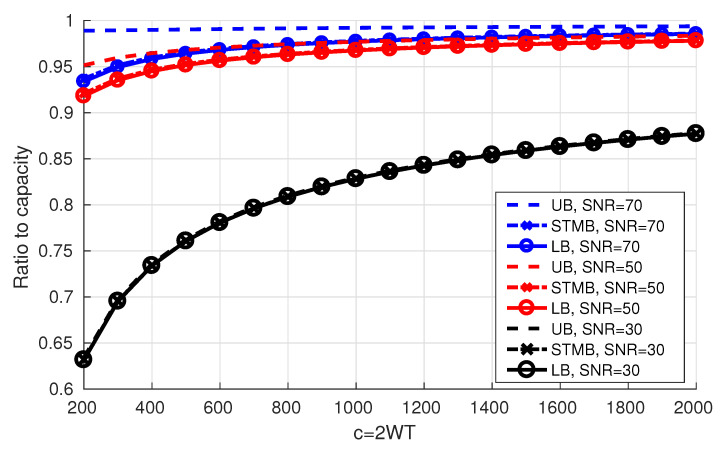
Upper and lower bounds on the rates.

Figure 10 shows the ratio of the obtained bounds to the Shannon capacity. For a fixed *c* and as 𝖲𝖭𝖱 increases, the bounds get relatively closer to capacity and the gap between the upper bound and the lower bound increases. In addition, for a fixed 𝖲𝖭𝖱, as *c* increases, the gap between the bounds decreases.

## 6. Possible Enhancements on the Bounds

Since the obtained upper and lower bounds on the rates (Figure 10) are very close for 𝖲𝖭𝖱 =30 dB, the bounds are tight and a good approximation for the optimal data rates is reached. However, the gap between the upper and lower bounds increases as the 𝖲𝖭𝖱 increases. For instance, the gap between the upper and lower bounds for c=200 and 𝖲𝖭𝖱 =70 dB is 5.5% of Shannon’s capacity, which means that one or both bounds are loose. In the following we present some possible improvements on the bounds.

### 6.1. A Tighter Upper Bound

To derive the upper bound, we ignored the interference due to other codewords and the obtained degrees of freedom surpassed 2WT for 𝖲𝖭𝖱 ∈ {50 dB,70 dB}. However, the results in the literature shows that the asymptotic dimension of the W-T space is 2WT, and thus adding the constraint that the the codewords must be time-limited will not increase the available degrees of freedom. So one can conclude that our upper bound on the degrees of freedom is not tight. One possible way to improve this upper bound is to force the degrees of freedom to be at most 2WT, and hence, *L* in constrained to be less or equal to 2WT in Equation (10). In other words, the normalized time-limited PSWFs $\frac{D\varphi_{c,m}\left(t\right)}{\sqrt{\lambda_{c,m}}}$ with m≥2WT will not be used to transmit data and their allocated power $P_{m}$’s are forced to be zero. The obtained upper bound will decrease and hence the gap between the upper and lower bounds will decrease.

**Figure 11:**
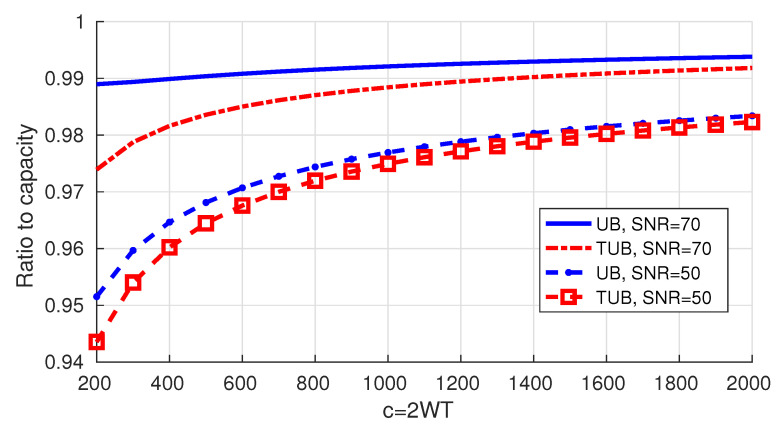
Tighter upper bound on the rates vs. the old ones.

In Figure 11 we present the numerical values for the tighter upper bound (TUB) in addition to those of the upper bound. When c=200, we note that the tighter upper bound achieves improvements of 0.8% and 1.5% for 𝖲𝖭𝖱 =50 dB and 𝖲𝖭𝖱 =70 dB, respectively.

### 6.2. Introducing a Guard Time or a Guard Band between the Codewords

The use of a guard time or a guard band is expected to decrease the effect of inter-codeword interference which drives towards increasing the achievable rates. On the other hand a portion of the time resources or frequency resources will not be used which decreases the data rates. Therefore, there must be an optimal guard time and an optimal guard band that maintain a good trade-off between the lost resources and the improvement of the inter-codeword interference.

Using a guard time $T_{G}$ reduces the upper bound to $R_{UB}\left(T_{G}\right)=R_{UB}\frac{T}{T+T_{G}}$, but introducing a guard band does not affect it. When it comes to the lower bound, deriving a closed form expression has proven to be difficult.

**Figure 12:**
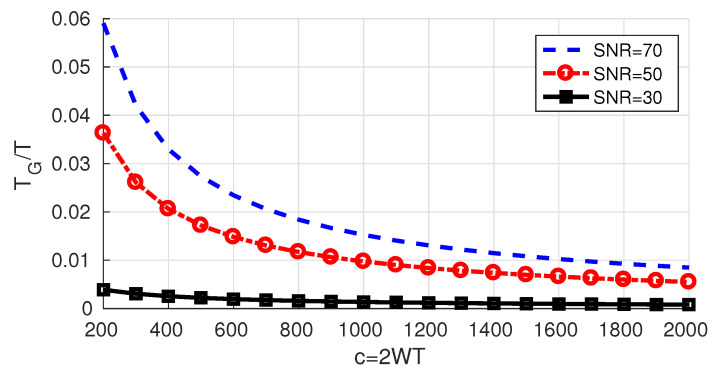
Upper bound on $\frac{T_{G}^{*}}{T}$ for different 𝖲𝖭𝖱 levels.

Nevertheless, if adding a guard time is beneficial, we can state that the optimal guard time $T_{G}^{*}$ must satisfy $R_{UB}\left(T_{G}^{*}\right)\geq R_{LB}$ where $R_{UB}$ and $R_{LB}$ are given by Equations (10) and (16) and hence $\frac{T_{G}^{*}}{T}\leq \frac{R_{UB}}{R_{LB}}-1$. In Figure 12, we draw the obtained upper bound on $T_{G}^{*}$. Naturally, it increases as the 𝖲𝖭𝖱 increases since the relative difference between the bounds increases as the 𝖲𝖭𝖱 increases.

## 7. Summary and Conclusions

In this work, we studied the maximal achievable rates and the available degrees of freedom when transmitting *T*-seconds time-limited codewords over a *W*-Hz band-limited AWGN channel. We made use of the prolate spheroidal wave functions to switch to discrete time and then applied the results by Polyanskiy. We derived the upper bound by ignoring the interference due to other codewords, and we derived lower bounds by deriving upper bounds on the inter-codeword interference. The derived bounds were found to be tight for for low values of 𝖲𝖭𝖱/*W* (for example W=1 KHz and 𝖲𝖭𝖱 =30 dB or equivalently W=100 KHz and 𝖲𝖭𝖱 =50 dB). However, the gap between the bounds increases as this 𝖲𝖭𝖱/*W* ratio increases.

When it comes to the available degrees of freedom, the numerical results showed that the potential decrease from 2WT is upper bounded by a logarithmic function of 2WT and hence the relative reduction is asymptotically negligible.

Based on the results of this work, one can approximate the achievable data rates by

$$\begin{array}{cc} R(\epsilon ,P,c)= & \frac{L}{T}\log_{2}\;\;\left[1+\frac{PT}{N_{0}L}\right]-\frac{\sqrt{2L}}{T}\sqrt{V_{1}\;\;\left[\frac{PT}{N_{0}L}\right]}Q^{-1}\left(\epsilon \right)+\frac{1}{2T}\log_{2}\left(2L\right), \end{array}$$

where $V_{1}\left(\theta \right)=\frac{\theta }{2}\frac{\theta +2}{{\left(\theta +1\right)}^{2}}\log_{2}^{2}e$ and *L* is in the range $c-1.35\log_{2}\left(c\right)+3.25\leq L\leq c$. This approximation is guaranteed to be between the derived bounds, and hence is a “good” one for low values of 𝖲𝖭𝖱/W.

## Appendix A. Properties of αc,l,mk,h

The inner product between the $l^{th}$ normalized time-limited PSWF already shifted in time and frequency by kT-seconds and h2W-Hz respectively, and the band-limited version of the $m^{th}$ normalized time-limited PSWF is denoted in this document by αc,l,mk,h. By Equation (5) and using Parseval,

αc,l,mk,0=def〈Dφc,l(t−kT)λc,lej2πh2W(t−kT),λc,mφc,m(t)〉=〈Φc,lD(f−h2W)e−j2πkTf,Φc,mD(f)rectf2W〉=〈Φc,lD(f−h2W)e−j2πkTfrectf2W,Φc,mD(f)〉.

**Property** **A1.**
*The coefficients αc,l,mk,h satisfy*

αc,l,mk,h=jm−lλc,mλc,lαc,m,l−h,−k.

**Proof.** This can be readily obtained by noting that by (4)

αc,l,mk,h=〈Dφc,l(t−kT)λc,lej2πh2W(t−kT),λc,mφc,m(t)〉=〈Φc,lD(f−h2W)e−j2πkTfrectf2W,Φc,mD(f)〉=〈jlT2Wφc,lT2W(f−h2W)e−j2πkTfrectf2W,jmT2Wφc,mT2Wf〉=〈jl−mT2Wφc,lT2W(f−h2W),φc,mT2Wfrectf2Wej2πkTf〉

By a change of variable ν=Tf/2W,

αc,l,mk,h=jl−m〈φc,l(ν−hT)φc,m(ν)rectνTej2πk2Wν〉=jl−m〈φc,l(ν)Dφc,m(ν+hT)ej2πk2W(ν+hT)〉=jl−mλc,mλc,l〈Dφc,m(ν+hT)λc,me−j2πk2W(ν+hT),λc,lφc,l(ν)〉=jm−lλc,mλc,lαc,m,l−h,−k.

□

### Upper Bounds

Various upper bounds on the magnitude of αc,l,mk,h may be found using Cauchy Schwarz’s inequality.

**Property** **A2.**
*The magnitude of αc,l,mk,h is upperbounded by*

(A1) |αc,l,mk,h|2≤λc,m∫(2k−1)T/2(2k+1)T/2|φc,m(t)|2dt

(A2) |αc,l,mk,h|2≤λc,m∫(−2h−1)T/2(−2h+1)T/2|φc,l(t)|2dt

(A3) |αc,l,mk,h|2≤∫(−2k−1)T/2(−2k+1)T/2|gh,l(t)|2dt

(A4) |αc,l,mk,h|2≤λc,mλc,l∫(2h−1)T/2(2h+1)T/2|g−k,m(t)|2dt

*where*

$$g_{h,l}\left(t\right)\overset{\mathrm{def}}{=}\left[\frac{D\varphi_{c,l}\left(t\right)}{\sqrt{\lambda_{c,l}}}\;e^{j2\pi h\;2Wt}\right]*2W\;\left(2W\;t\right)\;\;G_{h,l}\left(f\right)\overset{\mathrm{def}}{=}\Phi_{c,l}^{D}\left(f-h\;2W\right)\;\mathrm{rect}\;\left(\;\frac{f}{2W}\;\right).$$

Before proceeding to the proof, we specialize the bounds (A3) and (A4) to the values of h=0 and k=0 respectively. Using the fact that $g_{0,l}\left(t\right)=\left[\frac{D\varphi_{c,l}\left(t\right)}{\sqrt{\lambda_{c,l}}}\right]*2W\;\mathrm{sinc}\left(2W\;t\right)=\sqrt{\lambda_{c,l}}\;\varphi_{c,l}\left(t\right)$,

(A5) |αc,l,mk,0|2≤∫(−2k−1)T/2(−2k+1)T/2|g0,l(t)|2dt=λc,l∫(−2k−1)T/2(−2k+1)T/2|φc,l(t)|2dt

(A6) |αc,l,m0,h|2≤λc,mλc,l∫(2h−1)T/2(2h+1)T/2|g0,m(t)|2dt=λc,m2λc,l∫(2h−1)T/2(2h+1)T/2|φc,m(t)|2dt.

**Proof.** Bound (A1) is obtained by using Cauchy Schwarz,

αc,l,mk,h=〈Dφc,l(t−kT)λc,lej2πh2W(t−kT),λc,mφc,m(t)〉=〈Dφc,l(t−kT)λc,lej2πh2W(t−kT),λc,mφc,m(t)rectt−kTT〉|αc,l,mk,h|2≤λc,mφc,m(t)rectt−kTT2Dφc,l(t−kT)λc,l2=λc,m∫(2k−1)T/2(2k+1)T/2|φc,m(t)|2dt.

When it comes to (A2), using Property A1,

|αc,l,mk,h|2=λc,mλc,l|αc,l,mk,h|2≤λc,m∫(−2h−1)T/2(−2h+1)T/2|φc,l(t)|2dt.

Bound (A3) may be derived as follows:

αc,l,mk,h=〈Φc,lD(f−h2W)e−j2πkTfrectf2W,Φc,mD(f)〉=〈Φc,lD(f−h2W)rectf2W,Φc,mD(f)ej2πkTf〉=〈Gh,l(f),Φc,mD(f)ej2πkTf〉=〈gh,l(t)Dφc,m(t+kT)λc,m〉=〈gh,l(t)rectt+kTT,Dφc,m(t+kT)λc,m〉,

and hence, by Cauchy Schwarz

|αc,l,mk,h|2≤∫(−2k−1)T/2(−2k+1)T/2|gh,l(t)|2dt.

Using Property A1, bound (A4) is readily obtained

|αc,l,mk,h|2=λc,mλc,l|αc,m,l−h,−k|2≤λc,mλc,l∫(2h−1)T/2(2h+1)T/2|g−k,m(t)|2dt.

□

When it comes to sums over one index, one can immediately obtain the following inequalities.

**Corollary** **A1.**
*The following bounds are directly obtained from the respective bounds derived above.*

(A7) ∑k∈ℤ*|αc,l,mk,h|2≤λc,m(1−λc,m)

(A8) ∑h∈ℤ*|αc,l,mk,h|2≤λc,m(1−λc,l)

(A9) ∑k∈ℤ*|αc,l,mk,h|2≤∥gh,l(t)∥2−∫−T/2T/2|gh,l(t)|2dt≤∥gh,l(t)∥2

(A10) ∑h∈ℤ*|αc,l,mk,h|2≤λc,mλc,l∥g−k,m(t)∥2−∫−T/2T/2|g−k,m(t)|2dt≤λc,mλc,l∥g−k,m(t)∥2

(A11) ∑k∈ℤ*|αc,l,mk,h|2≤λc,l(1−λc,l)

(A12) ∑h∈ℤ*|αc,l,mk,h|2≤λc,m2λc,l(1−λc,m).

Finally, considering sums over the two indices *k* and *h*, we note by Plancherel that

$$\sum_{h\in {\mathbb{Z}}^{*}}\;∥g_{h,l}{\left(t\right)∥}^{2}=\sum_{h\in {\mathbb{Z}}^{*}}\;{∥G_{h,l}\left(f\right)∥}^{2}\;=\;\sum_{h\in {\mathbb{Z}}^{*}}\int_{(2h-1)W}^{(2h+1)W}\;{\left|\Phi_{c,l}^{D}\left(f\right)\right|}^{2}df=\left(1-\lambda_{c,l}\right),$$

and hence bounds (A9) and (A10) imply

(A13) ∑h∈ℤ*∑k∈ℤ*|αc,l,mk,h|2≤(1−λc,l)

(A14) ∑h∈ℤ*∑k∈ℤ*|αc,l,mk,h|2≤λc,mλc,l(1−λc,m).
